# Use of a toolbox of tailored evidence-based interventions to improve children’s physical activity and cardiorespiratory fitness in primary schools: results of the ACTIPROS cluster-randomized feasibility trial

**DOI:** 10.1186/s12966-023-01497-z

**Published:** 2023-08-18

**Authors:** Berit Brandes, Louisa Sell, Christoph Buck, Heide Busse, Hajo Zeeb, Mirko Brandes

**Affiliations:** 1 Department of Prevention and Evaluation, Leibniz Institute for Prevention Research and Epidemiology - BIPS, Achterstraße 30, 28359 Bremen, Germany; 2 Faculty 11 – Human and Health Sciences, University of Bremen, Bremen, Germany

**Keywords:** Physical activity, School-based health promotion, Toolbox, Accelerometry, Fitness, Primary schools, Health promoting schools

## Abstract

**Background:**

School-based physical activity (PA) promotion is usually conducted by providing one specific intervention. In contrast, the ACTIvity PROmotion via Schools (ACTIPROS) toolbox provides a set of twelve evidence-based PA interventions serving different domains of the Health Promoting Schools framework that primary schools can select according to their requirements. In this study, we tested the feasibility of the toolbox approach in primary schools.

**Methods:**

A two-arm cluster-randomized feasibility trial at primary schools (n = 5 intervention schools [IS], n = 5 control schools) located in the Federal State of Bremen, Germany, was conducted. Children’s habitual PA (GENEActiv, Activinsights Ltd.) and motor skills (Deutscher Motorik Test; DMT) were measured at the beginning (t0: Sept and Oct 2021) and at the end of the school year (t1: June and July 2022). Between Oct 2021 and July 2022, the ACTIPROS toolbox was implemented at IS. Teachers documented intervention choices and implementation within a short questionnaire (SIQ) at t1.

**Results:**

IS successfully implemented at least one intervention of the toolbox. In total, seven out of twelve possible interventions were selected. Two schools decided to replace an intervention with another during the trial. Results of the SIQ indicated that IS tended to choose similar interventions while implementation frequency was highly different. N = 429 students from two classes per school were recruited. The mean consent rate was 75.1% (n = 322). At t0 and t1, n = 304 (94.4%) and n = 256 (79.3%) of consented children took part in the DMT, respectively. The accelerometry sample included one class per participating school. At t0 and t1, n = 166 and n = 151 devices were handed out to students and n = 133 (80.1%) and n = 106 (70.2%) valid records could be retrieved, respectively. Linear mixed models showed an intervention effect of 15.5 min (95% CI: 4.5; 26.6) in children’s daily MVPA at IS between t0 and t1 compared to controls.

**Conclusions:**

All IS were able to implement at least one intervention from the toolbox, and unsuitable interventions were successfully replaced in a timely manner, highlighting the feasibility of implementing the ACTIPROS toolbox. Good consent rates for accelerometer and motor skills data were achieved. Results indicate a substantial increase in MVPA associated with the ACTIPROS toolbox and need to be tested in a larger sample.

**Trial registration:**

German Clinical Trials Register DRKS00025840.

**Supplementary Information:**

The online version contains supplementary material available at 10.1186/s12966-023-01497-z.

## Background

Physical inactivity is one of the four leading global health risks [1] for the most common diseases and leads to a reduction in life expectancy [2]. There is robust evidence on substantial health benefits for children who are more active and engage in higher volumes and intensities of physical activity (PA) [3]. Furthermore, a physically active lifestyle tracks into adulthood, therefore, the promotion of adequate PA at a young age is of great importance for the current and future health status [4]. Schools are key settings for health promotion as they provide high levels of reach and children spend much of their daytime at or in school [5]. PA promotion via schools normally offers one intervention to the school and many studies have been designed to investigate the effectiveness of one specific school-based intervention [6–8]. However, a growing understanding of the individual, social and organizational determinants influencing children’s activity behaviours contradicts this ‘one-size-fits-all’ approach and does not take setting-specific differences on the individual, social and environmental level of schools into account. Since substantial efforts were made to design and evaluate many different school-based PA interventions with diverse approaches, theoretical background, intensity and duration [9], it is possible to offer more than one intervention to schools. Therefore, in this feasibility study a toolbox approach containing twelve evidence-based PA promoting interventions has been applied and tested. Interventions in the ACTIvity PROmotion via Schools (ACTIPROS) toolbox include activities such as active breaks during and between school lessons, physical education sessions or active travel to school initiatives as well as interventions that encompass a combination of different components in line with a ‘whole school’ approach [10]. Whilst enormous efforts have been undertaken in developing and evaluating different school-based interventions to promote PA amongst children, crucial questions regarding the individual needs of different schools remain to ensure a successful implementation and sustainability [11]. One definition of ‘Implementation science’ which is a fairly new field of study “is the study of methods to promote the adoption and integration of evidence-based practices, interventions, and policies into routine health care and public health settings” [12]. In this regard, the general aim of the ACTIPROS study is to put a stronger emphasis on locally fit solutions for the promotion of PA at primary schools through the ACTIPROS toolbox approach. As a first step before considering a cluster randomized controlled trial (CRCT) to determine effectiveness it is important to explore whether the toolbox approach is feasible and that a potential RCT is sufficiently powered to detect a change in the target behaviour [13]. Furthermore, the piloting stage allows to identify any aspects of the toolbox that can be improved before progressing to a full trial. In line with the updated framework for developing and evaluating complex interventions by the Medical Research Council guidance [13], the design and conduct of this feasibility study were guided by the following research questions:

- Does a heterogeneous sample of local primary schools use the contents of the ACTIPROS toolbox?
- In case that an intervention fails, do schools successfully switch to a different intervention?
- Is it feasible to conduct a CRCT evaluation of the ACTIPROS toolbox that is designed to improve PA among primary school students?

To answer those research questions, the following objectives were formulated: to [1] give a detailed description of the development of the ACTIPROS toolbox and to evaluate the implementation compliance (e.g. number of applied sessions), [2] evaluate the recruitment rate of students, [3] estimate the likely accelerometer and motor skill data provision rate, [4] examine a potential change in PA and cardiorespiratory fitness (CRF) as well as possible side effects on motor abilities, and [5] estimate the sample size for an adequately powered CRCT to evaluate the ACTIPROS toolbox.

## Methods

### Study design and recruitment

**Fig. 1:**
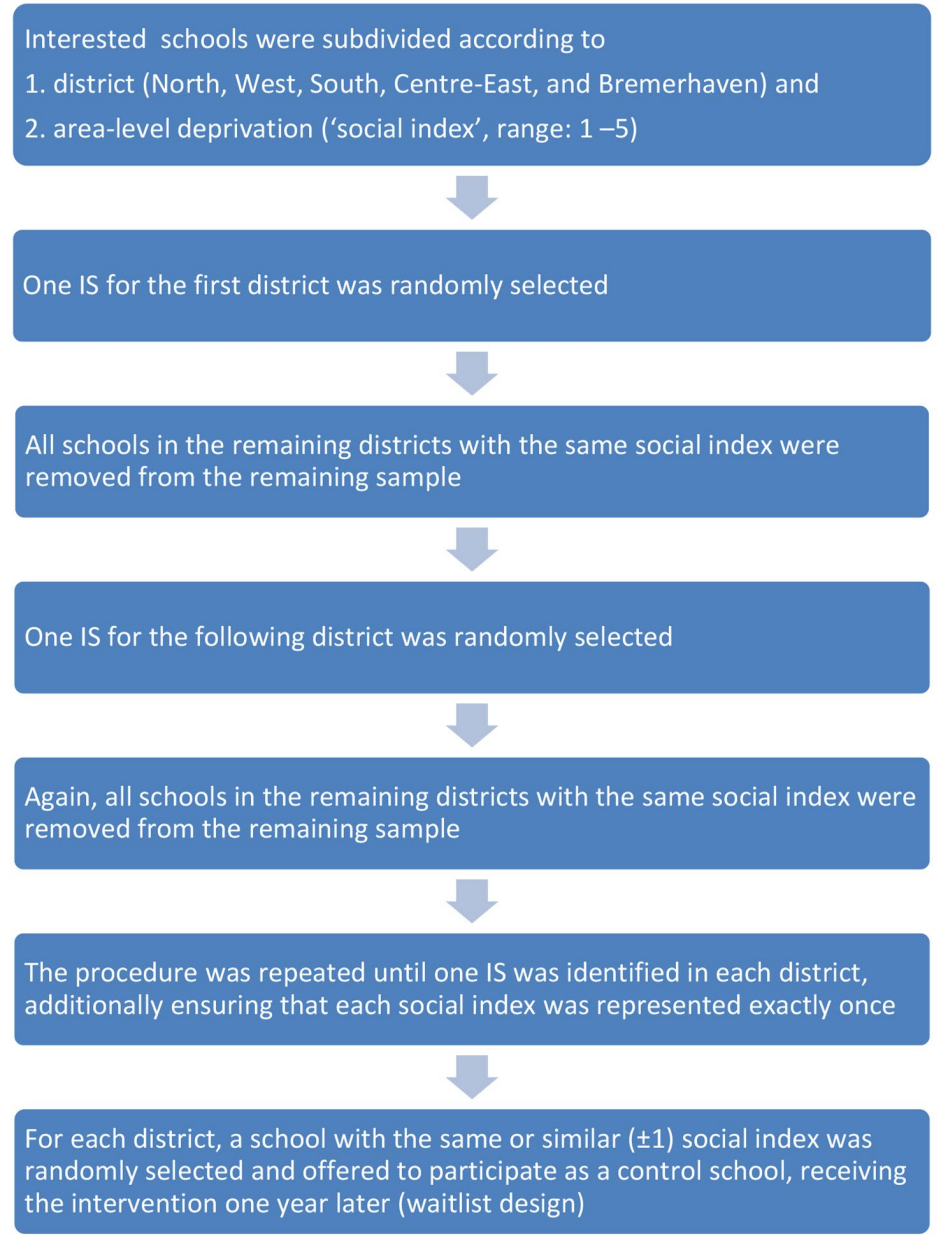
Selection process of participating intervention and control schools *Notes: IS = intervention school*

This study was a two-arm cluster-randomized feasibility trial, stratified by district and matched for area-level deprivation, with pre-/post measurements of habitual PA, CRF and motor skills conducted at ten primary schools, with schools as the unit of allocation. Trial design, analysis and interpretation was guided by the extension to randomised pilot and feasibility trials of the Consolidated Standards of Reporting Trials (CONSORT) 2010 statement and the ‘CONSORT 2010 checklist of information to include when reporting a pilot or feasibility trial [see Additional file 1]’ was used [14]. An embedded process evaluation was additionally undertaken, using qualitative data, but its results will be reported elsewhere. The recruitment procedures were developed in cooperation with local stakeholders. On average, ~ 25 pupils attend one class in Bremen primary schools. Based on previous experiences with research in Bremen primary schools, we aimed to approach 10 classes for the intervention and 10 classes for the control group to address a minimum of 200 pupils per group (= 400 pupils in total), which was deemed sufficient for the purposes of our feasibility study. It was further decided that the intervention and control classes should cover all five Bremen districts and that they should be matched by area-level deprivation index, ranging from 1 – highest social index, to 5 – lowest social index. Consequently, this study aimed at recruiting five intervention and five control schools while two classes per school were needed. In May 2021, letters were sent to all state-funded primary schools in Bremen and Bremerhaven (Germany) via e-mail by the Bremen Senator for Children and Education, inviting each school to register two classes for our study. Figure 1 illustrates the selection process among all primary schools that provided positive feedback and showed interest in participation.

The implementation of the random allocation sequence was generated by MB by using MS Excel 2013 random number generator (Mersenne-Twister algorithm). Given the procedure as described in Fig. 1, the random sequence automatically assigned the schools to intervention school or control school. Recruitment of pupils was done via letters to the parents of the selected classes which were provided in seven different languages (German, English, Turkish, Russian, Bulgarian, Arabic, Kurdish) and contained a study information, the baseline questionnaire, and the informed consent. Parents were asked to provide written informed consent for their child which children had to return at school and children gave verbal assent on the measurement day. Inclusion criteria at the individual level were apparently healthy children aged 6 to 10 years with written parental informed consent and data on motor skills from at least one timepoint. After the baseline assessment, we implemented our toolbox approach in both classes at each intervention school over the course of one school year. Classes at control schools followed their usual routine. All children received certificates for participating in our data collection at t0 and t1, respectively. Control schools were offered to select an intervention from the ACTIPROS toolbox at the end of the study. The Bremen University ethics committee approved the study (ref: 2021-17).

### Intervention: The ACTIPROS toolbox

Using a toolbox approach, the overall aim of ACTIPROS was to disseminate evidence-based PA and CRF promoting interventions via primary schools. Following the World Health Organisation Health Promoting Schools (WHO HPS) framework, the toolbox was envisaged to include a wide range of different interventions, including behavioural and environmental interventions, single and multicomponent interventions, and multilevel interventions e.g. those that include family or community components. The findings of our scoping review on evidence-based interventions for the promotion of PA and CRF in the school setting served as the basis for developing the ACTIPROS toolbox [15]. In total, data of N = 178 interventions were extracted and interventions were mapped according to the WHO HPS framework [15]. Among other outcomes, we extracted data on author’s conclusions on effectiveness regarding PA, sedentary behaviour and CRF outcomes [15]. All interventions that were deemed effective in regard to PA and CRF outcomes were considered for inclusion in the ACTIPROS toolbox (n = 73). In a second step, further criteria for the selection of interventions to promote PA and CRF among school children were determined via a three-round Delphi study with local stakeholders and international scientific experts [11]. Since the crucial question remains as to which interventions work in the ‘real world’ with the potential to be successfully implemented and sustained long-term, we deemed stakeholders’ views equally important as compared to the views of scientific experts to design an ACTIPROS toolbox that contains interventions that are considered suitable and practicable by both groups. After scoring the interventions according to the elaborated criteria, the interventions were rank-ordered. Subsequently, authors (n = 36) of the interventions were contacted top down, starting with the highest-ranked intervention, and were asked to provide their intervention materials and consent to include the intervention into the ACTIPROS toolbox. Fifteen of the contacted authors (42%) responded to our request. One author could not provide complete intervention materials and two interventions could solely be provided in languages other than English or German. Consequently, we received intervention materials of twelve evidence-based PA and CRF promoting interventions that could be adapted and, if necessary, translated into German language. Those twelve interventions were added to the toolbox version 1.0 containing a brief description in bullet point form for each intervention, so that schools can find suitable interventions quickly. The idea of ACTIPROS was to familiarize schools with the HPS framework and likewise with the complexity of different school-based interventions, to support schools in selecting suitable interventions for their individual needs and to accompany them during the implementation process. Therefore, the brief description in the toolbox contained information about the six different areas of the HPS framework covered by the respective intervention. In this feasibility trial, the research team guided each intervention school in working with the toolbox and provided the adapted and translated intervention materials of the twelve original interventions. For further information on the individual interventions, please refer to the references of the original publications (Table 1). For better reporting of interventions, we included the ‘Template for intervention description and replication (TIDieR) checklist [see Additional file 2]’ [16] but in case of our highly flexible toolbox approach and due to space constraints, we could not describe all included interventions in detail and had to refer to the original investigations of the included interventions. Through regular telephone contacts, researchers were quickly informed about implementation problems and assisted, if necessary, to switch to another intervention. Main characteristics of the included interventions in the ACTIPROS toolbox v1.0 are summarized in Table 1. Further information on the toolbox can be found on the project’s website: www.actipros.de. This feasibility study tested the usability of the German version of the ACTIPROS toolbox. Future plans also foresee an English version that can be used internationally.

**Table 1:** Main characteristics of the evidence-based PA and CRF promoting interventions included in the ACTIPROS toolbox v1.0

Name of intervention [German title in ACTIPROS toolbox v1.0]	HPS sections	Intervention duration	Intervention content	Targeted outcome(s)	Intervention provider	Material requirement	Estimated costs	Sustainability	Feasibility
Walking bus program [Der laufende Schulbus] (13)	LPC	5 weeks	Children walk together to school accompanied by parents of volunteers	PA	Parents, volunteers	Low	Low	High	Easy
Bicycle trains, cycling and physical activity [Der Fahrrad-Zug zur Schule] (14)	LPC	arbitrary	Children got to school together by bike accompanied by parents or volunteers	PA	Parents, volunteers	Low to high	Low to high	High	Easy
Physical activity and skills intervention (SCORES) [Gemeinsam Fit] (15)	LPC, HSP PSE, SSE	1 school year	Workshops for teachers and students; movement tasks for students and family member, establishment of a school committee; guidelines for PA promotion; strategies to improve cooperation between the school and the community	PA, CRF	Teachers	High	Medium to high	Medium	Medium
The Daily Mile [Die tägliche Meile] (16)	SSE	arbitrary	children go outside during regular classroom time for 15 min and exercise at a self-selected pace	PA, SB, CRF	Teachers	None	None	High	Easy
Bizzy Break! [Bewegte Pause] (17)	HSE	10 weeks	10 min of classroom PA breaks per day supported by a student poster, teacher notes and a music CD	PA	Teachers	Low	Low	High	Easy
Physical activity, children and the environment (PACE) [Aktiver Pausenhof] (18)	HSP, PSE	16 weeks	Restriction of sedentary behaviour to a maximum of 5 min during breaks, or 10 min during lunch break, extension of break times by at least 5 min	PA, SB	Teachers	High	High	Medium	Easy
Food & Fun After School [Bewegungsspiele nach der Schule] (19)	LPC	24 weeks	active play, creative learning and hands-on activities during regular afternoon care	PA	After-school personnel	Low	Low	High	Easy
Kid’s Choice Program [Sternchen für Bewegung] (20)	LPC, SSE	36 weeks	reward points for PA during breaks, reward days to exchange points for small prizes	PA	Parents, teachers and volunteers	Medium	Medium	Medium	Easy
GoKids [Fitnessstunde im Studio] (21)	LPC	10 weeks	three gym sessions a week after regular school hours, joint warm-up, fitness parcours in small groups	PA	Fitness trainer	Medium	Medium	Medium	Medium
Lunchtime Enjoyment Activity and Play [Bewegung, Spiel und Spaß] (22)	PSE	32 weeks	various objects from everyday life (e.g. cardboard, pipes, tires, ropes) are placed within the schoolyard as play materials	PA	Teachers	Medium	Low	Medium	Easy
Be smart. Join in. Be fit. [Fitness für Kids] (23)	HSE	40 weeks	two additional school hours weekly conducted by qualified trainers; exercise program with extensive motor training and a high amount of MVPA, workshop for teachers	CRF	Teachers, qualified trainers	Low	Medium	High	Easy
APPLE Schools [Bewegte Schule] (24)	HSP, LPC, SHS	arbitrary	Health mediators develop an action plan to promote PA at school, PA homework for students and their parents	PA	Health mediators	Low	Low	Medium	Easy

Notes LPC: links with parents and community; HSP: healthy school policies; SHS: Access to school health services; HSE: health skills and education; PSE: physical school environment; SSE: social school environment; PA: physical activity; SB: sedentary behaviour; CRF: cardiorespiratory fitness

### Measures

#### Implementation process

To explore whether a heterogeneous sample of local primary schools was able to use the ACTIPROS toolbox, we tracked the implementation of interventions over the course of one school year at each intervention site. Members of the research team were in contact with each intervention site over the course of the implementation period and documented the implementation process. Through personal and telephone contact schools reported their intervention choices, implementation issues and changes to the initial planning while working with the toolbox. Complementary, at t1, teachers of intervention classes were asked to fill out a short implementation questionnaire (SIQ) where they had to document intervention choices, for how long each intervention was implemented and how often intervention sessions took place. Teachers were asked to return the SIQ either electronically or via mail and up to three reminders were send to teachers by the research staff between July and September 2022.

#### Physical activity and motor skill data

In order to assess the feasibility of collecting accelerometer and fitness data and the likely change in MVPA and CRF that could be expected from taking part in the program, all participating children took part in the German Motor Ability Test (DMT 6–18) and half of the participants (one class per school) were asked to use a wrist-worn GENEActiv accelerometer (Activinsights Ltd, Kimbolton, UK) for seven consecutive days, 24 h per day, at t0 (Sept and Oct 2021) and t1 (June and July 2022), respectively.

The DMT 6–18 [17] is a motor ability test recommended by the German Society of Sport Science to test the general fitness of children between the age of 6 to 18 years. This test battery includes eight fitness indicators (6-minute run, sit-ups, push-ups, broad jump, 20-meter sprint, jumping sideways, balancing backwards, stand and reach). The items are covering multiple motor abilities, such as leg strength and coordination, as well as flexibility [17]. It is a frequently used test, particularly in German primary schools. DMT data collection took place at both time points at each school’s gym. Due to space constraints, the 20 m sprint could not be assessed in one school (control group). Furthermore, children were able to voluntarily decline participating in any of the individual test components since the DMT does not foresee calculation of a total score.

The GENEActiv accelerometer includes a triaxial acceleration sensor (ADXL345) with a ± 8-g dynamic range, is light weight and waterproof and has been shown to provide valid estimates of PA intensities among children [18]. All accelerometers were initialized according to the manufacturer’s guidelines and operated with 100 Hz. Children were instructed to wear the accelerometer at their non-dominant wrist on seven consecutive days and to not remove it while sleeping, showering or bathing. Non-wear time was estimated on the basis of the standard deviation and value range of each accelerometer axis, calculated for moving windows of 60 min with 15-minute increments [19, 20]. By using a 60-minute time window, the method aims to detect periods of monitor non-wear time lasting for more than 1 h which are the periods that would most impact summary measures [19]. For the analysis of a potential effect of the toolbox on children’s MVPA, only data of children with a wear time of at least 16 h per day on at least 4 days were included in the analysis as previously outlined by Antczak et al. [21]. Cut-points for the non-dominant wrist proposed by Phillips et al. [18] were used to estimate mean daily minutes of MVPA.

#### Weight and height

At t0 and t1, children’s height was measured to the nearest 1 cm using a stadiometer (Seca® type 213 stadiometer, Invicta Plastics Ltd., Leicester, UK) and children’s body weight was measured to the nearest 0.1 kg (Tanita BC 420-MA, Tanita Corporation, Tokyo, Japan). Children were then classified as underweight/normal or overweight/obese according to age- and sex-specific cut-offs derived from percentile curves by Cole and Lobstein [22].

#### Demographics

Parental education and children’s sex, age, and migration background were assessed at baseline by a parental (or legal guardians) questionnaire. This questionnaire was provided in seven different languages and was handed out by the teachers to all participating children. Children were asked to give the questionnaire to their parents and to return the completed questionnaire at school and to give it back to their teacher. The research team collected all completed questionnaires at schools. If information on education was available from both parents/legal guardians, parental education was classified into low, medium, and high according to Lampert et al. [23], otherwise it was set to missing. Data on migration background was compiled based on information on the country of birth and the nationality of both parents. Children classified as having a two-sided migration background had parents who both had immigrated to Germany and/or parents who were not German citizens; children classified as having a one-sided migration background had one parent that had immigrated to Germany and/or did not hold German citizenship [24]. If information on the country of birth or the nationality of one parent was not available, migration background of the child was set to missing.

### Statistical analysis

This trial aimed at assessing the feasibility of data collection and information on the implementation of a toolbox with evidence-based PA and CRF promoting interventions, therefor, the analyses focused on addressing key issues of feasibility rather than estimating group differences in outcomes. To be included in the final analysis, students were required to have data on at least one motor skill item on at least one timepoint as well as information on age and sex. As the study population consisted of different age groups, age and gender standardized z-scores based on normative data from [17] were calculated for each motor skill item. To describe the study sample and changes over the course of one school year in primary and secondary outcomes, descriptive statistics, including means, standard deviations, frequencies and percentages, were calculated for both groups and time points. To estimate recruitment and data provision rates, descriptive summary statistics were calculated by school class and trial arm at baseline and follow-up, respectively. To estimate the impact of the toolbox on MVPA and CRF at the end of the school year, we used mixed effects linear regressions accounting for repeated measures including time effect, group effect and the intervention effect as interaction of time and group. Mixed linear models were conducted with the 6-min-run z-score and MVPA as dependent variables adjusted by group, sex, migrant status, highest parental education, and BMI z-score where the second model was further adjusted for valid wear time. Based on some evidence of a differentiated effect of PA promoting interventions for boys and girls on MVPA, we re-ran the second model stratified by sex. Statistical analyses were based on the intention-to-treat principle and results were reported at the level of individual children. Sensitivity analyses with complete case vs. intent-to-treat analyses were conducted. Since this was a feasibility study, point estimates and 95% confidence intervals were described and interpreted and p-values were not presented. Statistical analyses were conducted using SAS 9.4 (SAS Institute Inc, Cary, NC) and particularly the GLIMMIX procedure to estimate linear mixed models. Power calculations were conducted to determine the sample size required to detect changes in the two primary outcomes PA and CRF at the post-test assessments. All calculations were based on 80% and 90% power with alpha levels set at P ≤ 0.05. Our sample size calculation for the PA outcome was based on values from the Kinder-Sportstudie (KISS) [25] using a standard deviation of SD = 33 and an intraclass correlation coefficient of ICC = 0.08. For the CRF outcome, we used the standard deviation of SD = 130 from the normative data of the DMT [17] and the intraclass correlation coefficient (ICC = 0.03) from the KISS study [25]. Potential sample sizes for a future trial were calculated in R (version 4.2.1) [26] using the CRTsize package (version 1.2) [27].

## Results

### Selection and implementation of interventions from the ACTIPROS toolbox

The selection and implementation process of the ACTIPROS toolbox is documented in Table 2. Seven out of twelve interventions included in the ACTIPROS toolbox were chosen by the ten intervention classes while each intervention class was able to implement at least one intervention during the study phase. At two schools, two interventions were terminated prematurely and were replaced by another intervention, in both cases with the help of the research team. A third intervention had to be cancelled due to restrictions following the SARS-CoV-2 pandemic. Seven out of ten intervention classes (70%) filled out the SIQ. Data from the SIQ confirmed that each reporting intervention class implemented up to three toolbox interventions between November 2021 and July 2022. One teacher reported that he/she continued implementing the toolbox until the end of September 2022. Intervention classes tended to choose similar interventions from the toolbox (Table 2). Therefore, the average duration of intervention implementation and frequency of implementation for the three most frequently implemented interventions are reported here. In first place, five intervention classes implemented “The Daily Mile” with a mean duration of 22 weeks and a mean frequency of 32 intervention sessions (range: 8 to 81). The second most frequently chosen intervention was “Bizzy Break!” (mean duration: 15 weeks) with a mean frequency of 46 intervention sessions (range: 20 to 72). Three intervention schools implemented “Be smart. Join in. Be fit.“ with mean duration of 15 weeks while on average five intervention sessions took place (range: 2 to 9) (Table 2).

**Table 2:** Documentation of the selection and implementation process at intervention classes

Name of intervention [German title in ACTIPROS toolbox v1.0] ¥	Total times selected (n)	Reasons for inclusion	Reasons for exclusion	Abandoned (Y/N)	Reason for termination	Mean duration (in weeks)	Average number of sessions (min; max)
**Walking bus program [Der laufende Schulbus] (13)**	2	Low effort	Parental involvement difficult, high traffic volume	Y	Parents did not participate	-	-
**Bicycle trains, cycling and physical activity [Der Fahrrad-Zug zur Schule] (14)**	2	Low effort	Parental involvement difficult, high traffic volume, few bicycle skills	Y	Parents did not participate	-	-
Physical activity and skills intervention (SCORES) [Gemeinsam Fit] (15)	-	-	High effort	-		-	-
**The Daily Mile [Die tägliche Meile] (16)**	5	Low effort, outdoors	Time constraints	N		22	32 (8; 81)
**Bizzy Break! [Bewegte Pause] (17)**	4	New content, manageable time required, easy to implement		N		15	46 (20; 72)
Physical activity, children and the environment (PACE) [Aktiver Pausenhof] (18)	-	-	-	-		-	-
Food & Fun After School [Bewegungsspiele nach der Schule] (19)	-	-	No afternoon service	-		-	-
Kid’s Choice Program [Sternchen für Bewegung] (20)	-	-	Reward system	-		-	-
**GoKids [Fitnessstunde im Studio] (21)**	1	-	-	Y	Restrictions following the SARC-CoV-2 pandemic	-	-
**Lunchtime Enjoyment Activity and Play [Bewegung, Spiel und Spaß] (22)**	2	-	-	N		#	N/A*
**Be smart. Join in. Be fit. [Fitness für Kids] (23)**	3	No physical education teachers at school, external qualified trainers		N		15	5 (2; 9)
APPLE Schools [Bewegte Schule] (24)	-	-	-	-		-	-

Notes: ¥ selected interventions are marked in bold font * N/A = not applicable, this intervention did not foresee any sessions; # no information available due to missing short implementation questionnaires from intervention sites

### Overview of participating schools, consent rates and data provision

**Fig. 2:**
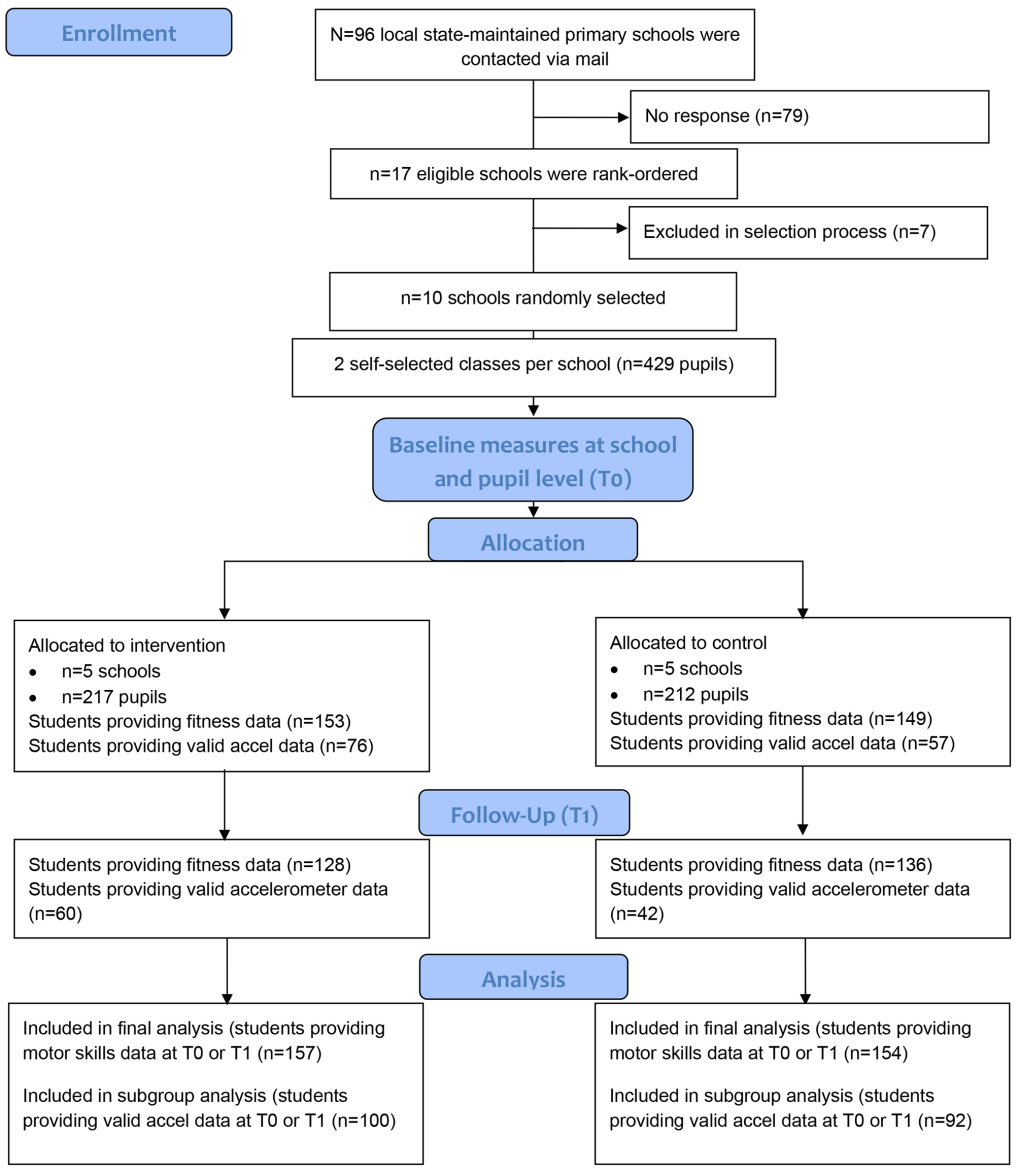
Flow diagram for ACTIPROS trial (based on CONSORT 2010 flow diagram)

School and student recruitment and retention are presented in Fig. 2. Recruitment and baseline data provision rates by school class are summarized in Table 3. The mean consent rate across all classes was 75.3% (n = 323) and varied between 37.5% and 100%. One parent in the control group withdrew his/her consent during the implementation phase resulting in a consent rate across all classes and in the accelerometry sample of 75.1% (n = 322) and 75.5% (n = 176), respectively. Of all consenting parents, n = 248 (77.0%) filled the baseline questionnaire. Parents in most cases preferred to fill the questionnaire in German language (n = 216, 89.3%). At t0 and t1, n = 304 (94.4%) and n = 256 (79.3%) of consented children took part in the DMT, respectively. At t0, N = 166 accelerometers devices were handed out to study participants. 164 (98.8%) of those devices were returned and n = 133 (80.1%) of the downloaded datasets fulfilled our wear time criteria of at least 16 h on at least 4 days. At t1, n = 151 devices were handed out to study participants, n = 143 (94.7%) of the devices were collected, and n = 106 records (70.2%) could be retrieved that fulfilled our wear time criteria.

**Table 3:** Participant recruitment, consent rate and baseline data provision rates by school class

School class	Arm	N pupils	Grade	Provided consent		Completed parental questionnaire		Motor skill data		Valid acc data	
				N	%	n	% of consented	n	% of consented	n	% of consented
1	I	24	3	18	75.0	17	94.4	18	100.0	N/A	N/A
2	I	24	3	15	62.5	11	73.3	13	86.7	12	80.0
3	I	22	1	22	100.0	16	72.7	21	95.5	14	63.6
4	I	24	1	6	25.0	4	66.7	4	66.7	N/A	N/A
5	I	22	4	22	100.0	10	45.5	22	100.0	17	77.3
6	I	19	3	12	63.2	7	58.3	11	91.7	N/A	N/A
7	I	16	1	11	68.8	6	54.5	10	90.9	N/A	N/A
8	I	19	4	16	84.2	13	81.3	16	100.0	14	87.5
9	I	23	3	22	95.7	16	72.7	22	100.0	19	86.4
10	I	24	1	20	83.3	17	85.0	18	90.0	N/A	N/A
11	C	24	4	9	37.5	5	55.6	9	90.0	6	60.0
12	C	24	4	11	45.8	10	90.9	10	90.9	10	90.9
13	C	21	2	16	76.2	13	81.3	16	100.0	N/A	N/A
14	C	19	3	13	68.4	11	84.6	13	100.0	11	84.6
15	C	28	2	25	89.3	14	56.0	24	100.0	N/A	N/A
16	C	18	3	13	72.2	9	69.2	12	92.3	6	46.2
17	C	22	1	22	100.0	19	86.4	20	90.9	N/A	N/A
18	C	20	3	15	75.0	13	86.7	13	86.7	10	66.7
19	C	18	3	16	88.9	15	93.8	15	93.8	N/A	N/A
20	C	18	3	18	100.0	16	88.9	17	94.4	14	77.8
All		429		322	75.1	242	75.2	304	94.4	N/A	N/A
Acc sample^1^		233		176	75.5					133	75.6

*Note*: ^*1*^*Accelerometry was only applied in every second class (n = 11), not in the full sample. Relative values refer to the accelerometry sample, not to the full sample*

*Acc: accelerometer; I: intervention group; C: Control group; N/A: not applicable*

### Participant characteristics

Descriptive characteristics of the study participants by trial arm at t0 and t1, respectively, are described in Table 4. The trial arms were well balanced at baseline in regard to the mean age of the participants with slightly differing proportions of boys, overweight children as well as families with high educational backgrounds (Table 4). In regard to the primary outcome, at baseline, children in the intervention group were slightly more active than children in the control (104.7 vs. 99.1 min per day of MVPA) and performed better in the 6-min run (16.3 vs. 15.2 laps).

**Table 4:** Descriptive data of study participants by trial arm at t0 and t1

Variable	T0				T1			
	CG		IG		CG		IG	
	n	MW ± SD / %	n	MW ± SD / %	n	MW ± SD / %	n	MW ± SD / %
Age (years)	149	8.4 ± 0.9	153	8.3 ± 1.3	136	9.1 ± 0.9	128	8.9 ± 1.3
Height (m)	149	1.3 ± 0.1	153	1.3 ± 0.1	136	1.4 ± 0.1	128	1.4 ± 0.1
Weight (kg)	149	32.6 ± 8.5	153	31.8 ± 8.8	136	35.5 ± 9.2	128	34.2 ± 9.6
Body-Mass-Index (kg/m²)	149	18.1 ± 3.4	153	17.7 ± 3.1	136	18.5 ± 3.5	128	17.9 ± 3.2
BMI category								
Normal weight/underweight	87	58.4	93	60.8	77	56.6	78	60.9
Overweight/obese	62	41.6	60	39.2	59	43.4	50	39.1
Sex								
Boys	78	52.3	86	56.2	73	53.7	72	56.3
Girls	71	47.7	67	43.8	63	46.3	56	43.8
Migration background								
No migration background	57	38.3	49	32.0	49	36.0	46	35.9
One-sided migration background	25	16.8	30	19.6	23	16.9	22	17.2
Two-sided migration background	37	24.8	35	22.9	36	26.5	29	22.7
Missing	30	20.1	39	25.5	28	20.6	31	24.2
Highest parental education								
Low	13	8.7	20	13.1	11	8.1	16	12.5
Medium	56	37.6	35	22.9	50	36.8	28	21.9
High	37	24.8	52	34.0	34	25.0	49	38.3
Missing	43	28.9	46	30.1	41	30.1	35	27.3
Physical fitness								
6-min run (laps)	148	15.2 ± 2.4	150	16.3 ± 2.4	136	15.4 ± 2.6	128	15.9 ± 2.8
6-min run (distance in m)	148	820.2 ± 131.2	150	879.0 ± 129.8	136	830.5 ± 141.5	128	860.3 ± 150.7
6-min run z-score	148	93.3 ± 9.7	150	98.2 ± 10.6	136	92.0 ± 10.5	128	94.5 ± 10.7
Physical Activity^1^								
MVPA (min per day)	57	99.1 ± 32.9	76	104.7 ± 32.4	42	96.1 ± 29.4	60	128.9 ± 42.5
Meeting guideline (≥ 90 min of MVPA per day)	35	61.4	48	63.2	22	52.4	51	85.0
Not meeting guidelines (< 90 min of MVPA per day)	22	38.6	28	36.8	20	47.6	9	15.0

*MVPA = moderate-to-vigorous physical activity*

^*1*^ *MVPA and adherence to PA guidelines by accelerometry was only assessed in every second class (n = 20), not in the full sample. Relative values refer to the accelerometry sample, not to the full sample*

### Changes in physical activity and motor skills

**Fig. 3:**
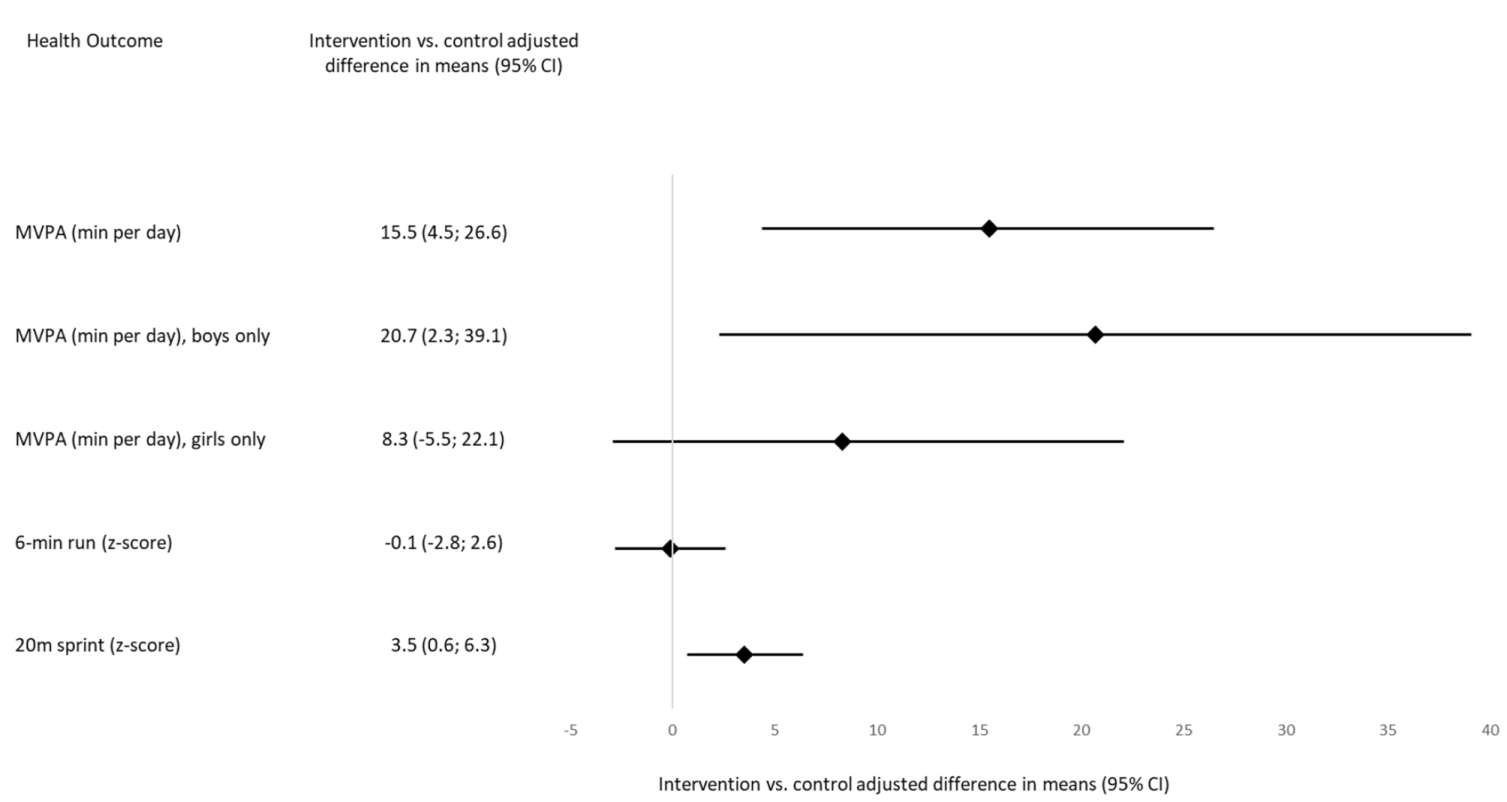
Forest plot of adjusted mean differences with 95% confidence intervals for outcomes (see additional files 3 and 4 for further details)

Linear mixed models showed an intervention effect of 15.5 min (95%CI: 4.5,26.6) in children’s daily MVPA at intervention schools between t0 and t1 compared to controls (Fig. 3), which was also reflected in a higher amount of children who met the PA guidelines at t1 compared to t0 in the intervention group (85% vs. 63.2%) while there was no such trend in the control group (52.4% vs. 61.4%) (Table 3). The adjusted mean difference in MVPA between the two groups was smaller for girls than for boys indicating that boys possibly benefitted more from the toolbox approach than girls did (Fig. 3). There was no trend towards an increase in CRF in favour of the intervention group (-0.1, 95% CI: -2.8; 2.6) (Fig. 3). At follow-up, children’s z-score in the intervention group increased by 3.5 points (95% CI: 0.6; 6.3) in the 20 m sprint compared to children in the control group (Fig. 3). Sensitivity analysis based on complete case analysis were very similar to the intent-to-treat analysis in the point estimates and 95% confidence intervals. For further details see ‘Adjusted between-group differences in physical activity and cardiorespiratory fitness at follow-up, complete case analysis’ and ‘Adjusted between-group differences in physical activity and cardiorespiratory fitness at follow-up, intent-to-treat analysis’ [Additional files 3 and 4].

For the power calculation of the MVPA and CRF outcome, we assumed a cluster size of n = 12 and n = 15 students per class, respectively. Power calculation resulted in a study sample of n = 552 with n = 46 clusters (i.e., classes) to detect an achievable between-group difference of 11 min per day of MVPA and a sample of n = 300 with n = 20 clusters (classes) to detect a between-group difference of 62 m on the 6-min run. Further details can be found in ‘Sample size calculation for a future definitive trial’ [Additional file 5].

## Discussion

To the best of our knowledge, this is the first study that systematically compiled evidence-based interventions to promote PA and CRF via primary schools into a toolbox and that piloted the feasibility of working with such a toolbox in the school setting. We obtained promising results in terms of recruitment, data collection and general acceptance of working with the ACTIPROS toolbox. A heterogeneous sample of local primary schools that was ranked and selected by a social index could be included. Intervention schools implemented up to three different interventions from the toolbox over the course of one school year. In case of any problems regarding intervention implementation, participating schools were able to successfully switch to another intervention.

Furthermore, we found that among the 10 intervention classes, seven out of twelve possible interventions were selected. Thus, schools in our sample had different interests and needs in the field of PA and CRF promotion, which could be met with a selection of different programs. Generally, intervention sites tended to choose smaller and less complex interventions in our feasibility study. While current scientific literature favours so called ‘complex interventions’ that address multiple levels at the same time [28], our impression was that schools need smaller interventions that can serve as ‘door openers’ and afterwards they might continue with more complex ones. It is also striking that two interventions that were abandoned were the only interventions that required parental participation. In addition, some schools excluded interventions that had foreseen parental participation as they saw little chance of success of these interventions. The scientific literature strongly recommends PA promoting interventions that include parental participation and parental participation is widely reported as an important factor regarding the effectiveness of PA promotion in children [8, 29, 30]. However, little is known on factors that foster parental participation and that can be effectively communicated with school staff. In summary, results of this study show that it is feasible a) to recruit students to participate in a CRCT examination of the ACTIPROS toolbox and b) to collect accelerometer and motor skills data at two timepoints in this target group. Last but not least, one of the most important questions remains whether individually positively evaluated interventions, put together as a toolbox, can also demonstrably increase children’s PA behaviour. This cluster-randomized feasibility study gives some initial results showing an intervention effect of 15.5 min of MVPA per day in favour of children from the intervention group. We regard this a promising finding and are therefore strongly interested in conducting an adequately powered trial CRCT with the aim to evaluate the effectiveness of our ACTIPROS toolbox .

### Strengths and limitations

One strength of this pilot study is that we tested the feasibility and acceptability of our ACTIPROS toolbox in a heterogenous sample of local primary schools. Recruitment of schools which was supported by the Bremen Senator for Children and Education went well and none of the participating school classes dropped out during the study period. Furthermore, we provided our parental study materials in seven different languages to avoid language barriers. Regarding participation in the DMT and provision of valid accelerometer data we received good response rates that are in line with those of similar studies while questionnaire data provision rates were lower in our study [31, 32]. One limitation is that due to moderate response rates for the parental questionnaires there was a lack of information on educational and migration background of some participating children. Also, in this cluster-randomized feasibility trial, stratified by district and matched for area-level deprivation, the two groups showed small differences in regard to baseline MVPA and CRF levels. Generalizability of our findings is limited due to the design and the specific contexts of our school sample. To further answer the question whether different schools can use the ACTIPROS toolbox and adapt it to their individual needs, future trials are needed to more broadly examine the feasibility and ultimately the effectiveness of the ACTIPROS toolbox approach in different contexts.

## Conclusions

In this study we successfully recruited 6 to 10-year-old pupils to participate in interventions from the ACTIPROS toolbox and the associated control arm. We were able to demonstrate the feasibility of collecting accelerometer and motor skills data at two timepoints. We also showed that the provision of a toolbox with different interventions for the promotion of PA and CRF based on a comprehensive scoping review and mapped according to the WHO HPS framework was well appreciated by school staff. All intervention classes from a heterogenous sample of local primary schools were able to implement at least one intervention from the ACTIPROS toolbox, and unsuitable interventions were successfully replaced in a timely manner. The data demonstrate that the ACTIPROS toolbox has the potential to positively affect children’s MVPA, but a future full trial is needed to provide reliable evidence. We estimated that for such a trial a sample of 552 children, recruited from 46 classes would be needed to detect an 11 min difference in daily MVPA.

### Electronic supplementary material

Below is the link to the electronic supplementary material.

Additional file 1: CONSORT checklist for pilot trials

Additional file 2: Template for intervention description and replication (TIDieR) checklist

Additional file 3: Adjusted between-group differences in physical activity and cardiorespiratory fitness at follow-up, complete case analysis

Additional file 4: Adjusted between-group differences in physical activity and cardiorespiratory fitness at follow-up, intent-to-treat analysis

Additional file 5: Sample size calculation for a future definitive trial

## Data Availability

The datasets used and/or analysed during the current study are available from the corresponding author on reasonable request.

## Abbreviations

CRF: Cardiorespiratory fitness
CI: Confidence interval
CONSORT: Consolidated Standards of Reporting Trials
ICC: Intracluster correlation coefficient
MVPA: Moderate-to-vigorous physical activity
PA: Physical activity
CRCT: Cluster randomized controlled trial
SD: Standard deviation
WHO: World Health Organization
HPS: Health Promoting Schools
