# Supplementary material for: Use of a toolbox of tailored evidence-based interventions to improve children’s physical activity and cardiorespiratory fitness in primary schools: results of the ACTIPROS cluster-randomized feasibility trial
Source: Int J Behav Nutr Phys Act. 2023 Aug 18;20:99. doi: 10.1186/s12966-023-01497-z (PMC10439638; doi:10.1186/s12966-023-01497-z)

CONSORTꢀ2010ꢀchecklistꢀofꢀinformationꢀtoꢀincludeꢀwhenꢀreportingꢀaꢀpilotꢀorꢀfeasibilityꢀtrial*ꢀ

ꢀ

Item

Reported

Section/Topic

No Checklist item

on page No

Title and abstract

Identification as a pilot or feasibility randomised trial in the title

1

1

a

b

1

Structured summary of pilot trial design, methods, results, and conclusions (for specific guidance see

CONSORT abstract extension for pilot trials)ꢀ

2-3

Introduction

Background and

objectives

Scientific background and explanation of rationale for future definitive trial, and reasons for randomised pilot

trial

2a

3-4

4

2

b

Specific objectives or research questions for pilot trial

Methods

Description of pilot trial design (such as parallel, factorial) including allocation ratioꢀ

Trial design

3a

5

Important changes to methods after pilot trial commencement (such as eligibility criteria), with reasons

3

b

NA

Participants

4a

Eligibility criteria for participants

6

4

b

c

Settings and locations where the data were collected

How participants were identified and consented

9

6

4

Interventions

Outcomes

5

The interventions for each group with sufficient details to allow replication, including how and when they were

actually administered

Completely defined prespecified assessments or measurements to address each pilot trial objective specified in

6-8, Table 1

6a

8-10

2

b, including how and when they were assessedꢀ

Any changes to pilot trial assessments or measurements after the pilot trial commenced, with reasons

6

b

c

NA

NA

5

If applicable, prespecified criteria used to judge whether, or how, to proceed with future definitive trial

6

Sample size

7a

Rationale for numbers in the pilot trial

7

b

When applicable, explanation of any interim analyses and stopping guidelines

NA

Randomisation:

Sequence

8a

8b

9

Method used to generate the random allocation sequence

Type of randomisation(s); details of any restriction (such as blocking and block size)

5

5

5

generation

Mechanism used to implement the random allocation sequence (such as sequentially numbered containers),

describing any steps taken to conceal the sequence until interventions were assigned

Allocation

concealment

mechanism


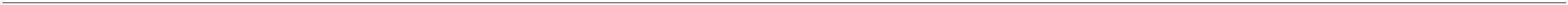

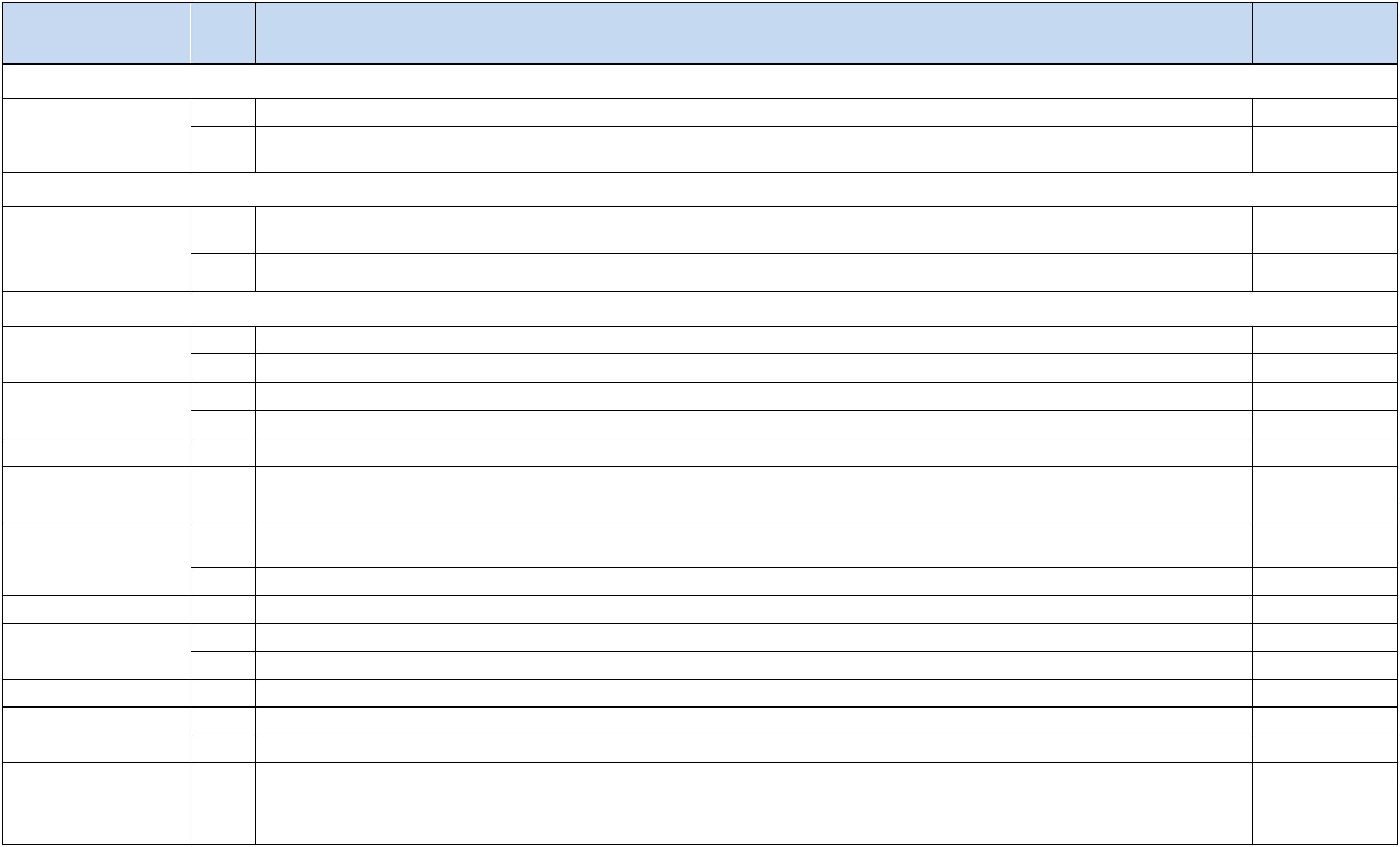

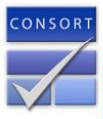


Implementation

Blinding

10

Who generated the random allocation sequence, who enrolled participants, and who assigned participants to

interventions

6

6

11a If done, who was blinded after assignment to interventions (for example, participants, care providers, those

assessing outcomes) and how

1

1b If relevant, description of the similarity of interventions

NA

Methods used to address each pilot trial objective whether qualitative or quantitative

Statistical methods

12

11-12

Results

For each group, the numbers of participants who were approached and/or assessed for eligibility, randomly

assigned, received intended treatment, and were assessed for each objective

Participant flow (a

diagram is strongly

recommended)

13a

Figure 1

13b For each group, losses and exclusions after randomisation, together with reasons

Figure 1

Recruitment

14a Dates defining the periods of recruitment and follow-up

5

1

4b Why the pilot trial ended or was stopped

NA

Baseline data

15

16

A table showing baseline demographic and clinical characteristics for each group

For each objective, number of participants (denominator) included in each analysis. If relevant, these numbers

Table 3

Table 3

Numbers analysed

should be by randomised group

For each objective, results including expressions of uncertainty (such as 95% confidence interval) for any

estimates. If relevant, these results should be by randomised group

Outcomes and

estimation

17

Table 4

Results of any other analyses performed that could be used to inform the future definitive trial

All important harms or unintended effects in each group (for specific guidance see CONSORT for harms)

Ancillary analyses

Harms

18

19

Table 5

N/A

N/A

1

9a If relevant, other important unintended consequences

Discussion

Limitations

Pilot trial limitations, addressing sources of potential bias and remaining uncertainty about feasibility

Generalisability (applicability) of pilot trial methods and findings to future definitive trial and other studies

20

18

18

Generalisability

Interpretation

21

22

Interpretation consistent with pilot trial objectives and findings, balancing potential benefits and harms, and

considering other relevant evidence

16-17

Implications for progression from pilot to future definitive trial, including any proposed amendments

2

2a

18

Other information

Registration

Protocol

23

24

25

Registration number for pilot trial and name of trial registry

Where the pilot trial protocol can be accessed, if available

3 (DRKS)

DRKS

20

Funding

Sources of funding and other support (such as supply of drugs), role of funders

Ethical approval or approval by research review committee, confirmed with reference number

2

6

6


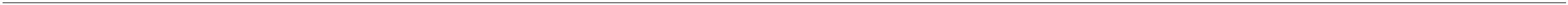

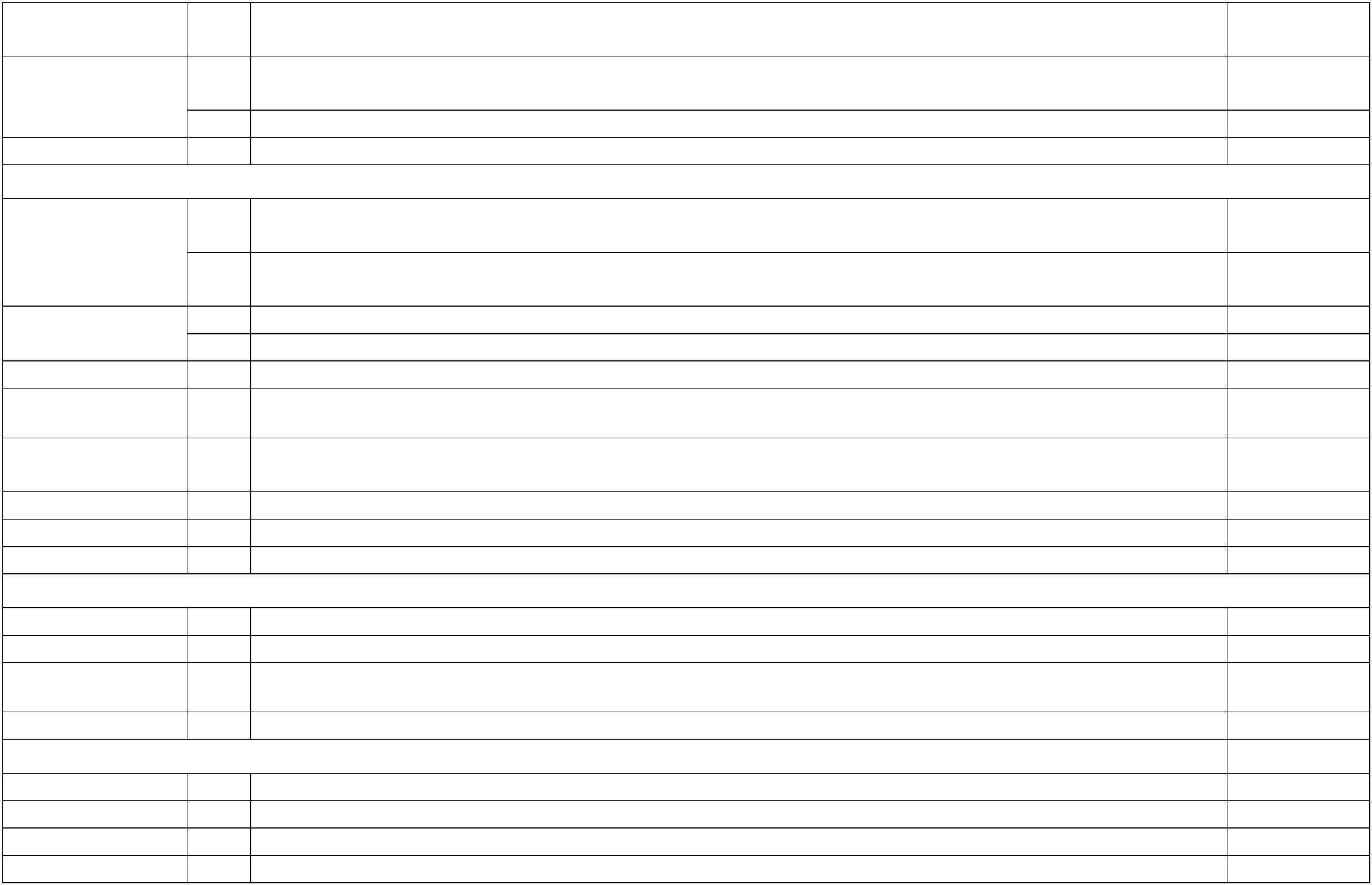


Citation: Eldridge SM, Chan CL, Campbell MJ, Bond CM, Hopewell S, Thabane L, et al. CONSORT 2010 statement: extension to randomised pilot and feasibility trials. BMJ. 2016;355.

*We strongly recommend reading this statement in conjunction with the CONSORT 2010, extension to randomised pilot and feasibility trials, Explanation and Elaboration for important

clarifications on all the items. If relevant, we also recommend reading CONSORT extensions for cluster randomised trials, non-inferiority and equivalence trials, non-pharmacological

treatments, herbal interventions, and pragmatic trials. Additional extensions are forthcoming: for those and for up to date references relevant to this checklist, see www.consort-statement.org.


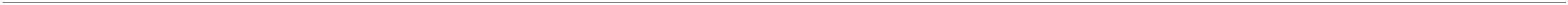

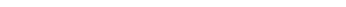

Supplement: Supplementary file 1 — Additional file 1: CONSORT checklist for pilot trials [file 12966_2023_1497_MOESM1_ESM.docx]
