# Supplementary material for: Use of a toolbox of tailored evidence-based interventions to improve children’s physical activity and cardiorespiratory fitness in primary schools: results of the ACTIPROS cluster-randomized feasibility trial
Source: Int J Behav Nutr Phys Act. 2023 Aug 18;20:99. doi: 10.1186/s12966-023-01497-z (PMC10439638; doi:10.1186/s12966-023-01497-z)
Supplement: Supplementary file 3 — Additional file 3: Adjusted between-group differences in physical activity and cardiorespiratory fitness at follow-up, complete case analysis [file 12966_2023_1497_MOESM3_ESM.docx]

Additional file 3 Adjusted between-group differences in physical activity and cardiorespiratory fitness at follow-up, complete case analysis

| Variable | Control | | Intervention | | Intervention vs. control adjusted difference in means (95% CI) |
| --- | --- | --- | --- | --- | --- |
|  | n | Mean ± SD | n | Mean ± SD |  |
| T0 | | | | | |
| MVPA (min per day) | 30 | 94.3±26.5 | 52 | 108.4±33.0 | - |
| Boys only | 13 | 100.2±29.7 | 30 | 119.0±31.2 |  |
| Girls only | 17 | 89.7±23.6 | 22 | 94.0±30.4 |  |
| 6-min run (z-score) | 130 | 92.9±10.1 | 123 | 98.2±10.7 | - |
| 20m sprint (z-score) | 100 | 97.1±10.1 | 124 | 96.2±10.3 | - |
| T1 | | | | | |
| MVPA (min per day) | 30 | 100.4±31.2 | 52 | 127.6±44.6 | 12.6 (0.7; 24.4)^b^ |
| Boys only | 13 | 105.4±30.2 | 30 | 141.6±44.8 | 15.7 (-3.6; 35.0) ^b^ |
| Girls only | 17 | 95.9±32.4 | 22 | 106.8±36.1 | 7.5 (-7.5; 22.4) ^b^ |
| 6-min run (z-score) | 130 | 91.8±10.5 | 123 | 94.5±10.7 | -0.2 (-3.0; 2.5)^a^ |
| 20m sprint (z-score) | 100 | 95.8±10.1 | 124 | 97.2±9.1 | 3.5 (0.6; 6.4) ^a^ |

Notes: ^a^ Adjusted for children’s age, sex, migration background, obesity status and parental education

^b^ Adjusted for children’s age, sex, migration background, obesity status, parental education and accelerometer wear time

MVPA: moderate-to-vigorous physical activity
