# Supplementary material for: Use of a toolbox of tailored evidence-based interventions to improve children’s physical activity and cardiorespiratory fitness in primary schools: results of the ACTIPROS cluster-randomized feasibility trial
Source: Int J Behav Nutr Phys Act. 2023 Aug 18;20:99. doi: 10.1186/s12966-023-01497-z (PMC10439638; doi:10.1186/s12966-023-01497-z)
Supplement: Supplementary file 4 — Additional file 4: Adjusted between-group differences in physical activity and cardiorespiratory fitness at follow-up, intent-to-treat analysis [file 12966_2023_1497_MOESM4_ESM.docx]

Additional file 4 Adjusted between-group differences in physical activity and cardiorespiratory fitness at follow-up, intent-to-treat analysis

| Variable | Control | | Intervention | | Intervention vs. control adjusted difference in means (95% CI) |
| --- | --- | --- | --- | --- | --- |
|  | n | Mean ± SD | n | Mean ± SD |  |
| T0 | | | | | |
| MVPA (min per day) | 57 | 99.1±32.9 | 76 | 104.7±32.4 | - |
| Boys only | 28 | 110.3±35.1 | 41 | 113.7±33.0 |  |
| Girls only | 29 | 88.3±27.1 | 35 | 94.2±28.6 |  |
| 6-min run (z-score) | 148 | 93.3±9.7 | 150 | 98.2±10.6 | - |
| 20m sprint (z-score) | 115 | 97.2±10.2 | 153 | 96.0±10.7 | - |
| T1 | | | | | |
| MVPA (min per day) | 42 | 96.1±29.4 | 60 | 128.9±42.5 | 15.5 (4.5; 26.6)^b^ |
| Boys only | 15 | 105.6±29.1 | 36 | 141.7±42.1 | 20.7 (2.3; 39.1) ^b^ |
| Girls only | 27 | 90.9±28.8 | 24 | 109.5±35.9 | 8.3 (-5.5; 22.1) ^b^ |
| 6-min run (z-score) | 136 | 92.0±10.5 | 128 | 94.5±10.7 | -0.1 (-2.8; 2.6)^a^ |
| 20m sprint (z-score) | 104 | 96.0±10.1 | 128 | 97.3±9.0 | 3.5 (0.6; 6.3) ^a^ |

Notes: ^a^ Adjusted for children’s age, sex, migration background, obesity status and parental education

^b^ Adjusted for children’s age, sex, migration background, obesity status, parental education and accelerometer wear time

MVPA: moderate-to-vigorous physical activity
