# Supplementary material for: Use of a toolbox of tailored evidence-based interventions to improve children’s physical activity and cardiorespiratory fitness in primary schools: results of the ACTIPROS cluster-randomized feasibility trial
Source: Int J Behav Nutr Phys Act. 2023 Aug 18;20:99. doi: 10.1186/s12966-023-01497-z (PMC10439638; doi:10.1186/s12966-023-01497-z)
Supplement: Supplementary file 5 — Additional file 5: Sample size calculation for a future definitive trial [file 12966_2023_1497_MOESM5_ESM.docx]

Additional file 5 Sample size calculation for a future definitive trial

| Between-arms difference | Power | N students (uninflated) | N students (inflated) | N classes |
| --- | --- | --- | --- | --- |
| MVPA (min per day) | | | | |
| 11 | 90 | 1.306 | 744 | 62 |
| **11** | **80** | **968** | **552** | **46** |
| 8 | 90 | 2.358 | 1.344 | 112 |
| 8 | 80 | 1.768 | 1.008 | 84 |
| 6-min run (m) | | | | |
| **62** | **90** | **422** | **300** | **20** |
| 62 | 80 | 338 | 240 | 16 |

The following parameters were used: cluster size for MVPA outcome = 12, cluster size for fitness outcome=15; ICC on MVPA = 0.08, ICC on CRF = 0.03; MVPA standard deviation = 33, 6-min run standard deviation = 130, 5% two-sided alpha and inflation to account for 43% and 29% of participants not providing verbal and written informed consent and valid accelerometer and participants not providing verbal and written informed consent and fitness data, respectively. Values in boldface represent the sample size values proposed for the primary outcomes in a future definitive trial.

MVPA: moderate-to-vigorous physical activity
